# Apexification of an Endodontically Failed Permanent Tooth with an Open Apex: A Case Report with Histologic Findings

**DOI:** 10.3390/medicina61020276

**Published:** 2025-02-06

**Authors:** Sundus Bukhary

**Affiliations:** Division of Endodontics, Department of Restorative Dental Science, College of Dentistry, King Saud University, P.O. Box 45347, Riyadh 13313, Saudi Arabia; sbukhary@ksu.edu.sa; Tel.: +966-1805-8240

**Keywords:** apexification, endodontic failure, open apex, histology, calcium silicate bioceramic

## Abstract

The management of an endodontic failure in a traumatized tooth with an open apex presents a major dilemma. The arrest of root growth after traumatic injury is a substantial consequence of pulpal necrosis. Conventional endodontic treatment procedures will be challenging by the resulting thin, fragile dentinal walls, which will impede the appropriate debridement and optimal apical sealing. Apexification is a well-established procedure that is designed to treat or preserve a tooth with an incomplete root apex and necrotic pulpal tissue by promoting the formation of a calcified apical barrier through the application of a bioactive substance at the root apex. The present case report demonstrates a successful apexification procedure for an endodontically failed permanent central incisor with an open apex with a two-year follow-up time. The clinical and radiographical results revealed the absence of signs and symptoms and the formation of hard tissue at the root apex. The tooth was extracted for prosthodontic reasons and processed for histologic examination. The histologic evaluation revealed dentin-like and cementum-like tissues formed at the apical barrier.

## 1. Introduction

Traumatic injuries to permanent teeth may result in damage to the periodontium, adjacent bone, and the neurovascular supply of the pulp. The outcome of the compromised pulp will be dictated by the natural balance between cellular ingrowth and bacterial infiltration, resulting in either sterile necrosis, infection-induced necrosis, revascularization, or regeneration of the injured pulp [1]. A significant consequence of developing pulp necrosis in a traumatized immature tooth is the cessation of root growth. This occurrence will result in thin, fragile dentinal walls, complicating appropriate debridement and optimal apical sealing with conventional endodontic treatment procedures [2,3].

The management of such cases is considered to be challenging for the dental professionals, necessitating different approaches. Traditionally, the apexification procedure served as a treatment modality to either induce the formation of an apical barrier or continue the development of an immature apex [4]. For an extended period of time, apexification entails the application of calcium hydroxide (Ca[OH]_2_) paste to achieve root-end closure, which was subsequently followed by root canal therapy [5]. This long-term therapy presents several disadvantages, such as challenges in patient follow-up, inconsistency in process of apical closure, and compromised tooth structure, which increases the risk of root fracture [6,7].

Subsequently, mineral trioxide aggregate (MTA), a calcium silicate-based hydrophilic cement, was introduced to the area of endodontics by Torabinejad and colleagues. This material demonstrated biocompatibility, induced odontoblastic development, exhibited antibacterial properties, possessed low solubility, and expanded upon setting; hence, MTA emerged as the preferred material for apexification by facilitating the placement of an artificial apical plug to encourage apical-end closure [8,9]. Nevertheless, the MTA possesses hydrophilic characteristics that necessitate moisture for the setting process, along with prolonged setting times extended up to 3 h and handling challenges, prompting the exploration of alternate materials [10]. Subsequent members of the calcium silicate-based materials were introduced to address these issues, including Biodentine^™^ (Septodont, Saint-Maur-des-Fosses, France), iRoot BP Plus (Innovative BioCeramix, Vancouver, BC, Canada), TotalFill^®^ BC RRM^™^ Putty (FKG Dentaire, Sàrl Le Crêt-du-Locle, Switzerland), among various other brands. These materials have decreased the setting time to an average of 9–12 min, hence eliminating the two-step obturation procedure [11,12]. Consequently, such materials were utilized in apexification situations.

Regenerative endodontic treatment (RET) is a treatment modality that has been implemented in recent years to address the condition of properly selected cases of immature permanent teeth with necrotic pulp. This treatment aims to revitalize the damaged tissues within the canal space and facilitate the maturation of the root as well as thickening the dentinal walls by hard tissue deposition [13]. RET is founded on a tissue bioengineering paradigm that incorporates four critical components: stem cells, scaffolds, bioactive growth factors, and disinfection, to achieve successful outcomes [14]. Despite the fact that RET was regarded an alternative treatment option for an infected immature tooth, numerous studies demonstrated a lack of consistency in the growth of root lengthening, thickening, and apical closure [15,16].

Apexification is a well-established treatment that has been shown to have favorable outcomes and consistent results, as evidenced by several clinical studies and case reports. The primary radiographic outcomes seen are the resolution of apical radiolucency, development of an apical barrier, and apical closure [17,18]. Histological studies of apexification procedures in human and animal models demonstrated the formation of newly mineralized tissue above the apical foramen, defined as either bone-like tissue, cementum-like tissue, or osteodentin tissue [19,20,21].

To our knowledge, there is limited histological evidence supporting the apexification treatment of an endodontically failed tooth. The present case describes the successful clinical and histological observations of an apexification procedure for an endodontically failed tooth with an open apex.

## 2. Case Presentation

A 24-year-old Caucasian female patient was referred to the Department of Endodontics at the College of Dentistry, King Saud University, Riyadh, Saudi Arabia, to assess the right maxillary central incisor. The patient’s chief complaint was the presence of mild-to-moderate pain during biting and discoloration on her upper front teeth. The patient had a history of trauma to the anterior maxillary region 10 years ago, during which she underwent root canal treatment at a private clinic. The patient has no history of any systemic disease, and according to the American Society of Anesthesiologists (ASA) classification, she is class ASA I.

**Figure 1:**
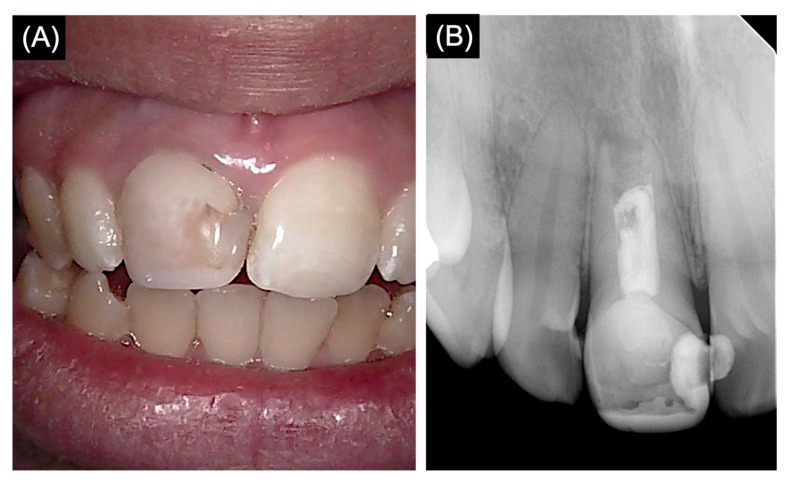
(**A**) Clinical photograph of tooth number 11. (**B**) Preoperative periapical radiograph of tooth number 11.

A clinical examination of the right maxillary central incisor (#11) revealed a defective tooth-colored restoration and mild crown discoloration compared to the adjacent teeth (Figure 1A). The pulp testing, which involved applying Endo-Frost (Coltène/Whaledent GmbH+ Co. KG, Langenau, Germany) with a cotton pellet and using an electric pulp tester (Analytic Technology, Redmond, WA, USA), revealed no response. Percussion and palpation recorded mild tenderness and pain; the tooth showed no mobility, and periodontal probing depths were within normal limits.

The preoperative periapical radiograph revealed an inadequate root canal filling that was short of the apex, accompanied by defective tooth-colored restoration (Figure 1B). The apical region of the root exhibited a short root with a blunderbuss canal and an open apex, along with slight apical radiolucency. Based on clinical and radiographic findings, the endodontic diagnosis revealed a previously treated tooth with symptomatic apical periodontitis.

Subsequent to a thorough discussion of the treatment options with the patient, the options presented include: an endodontic approach followed by the placement of a post/core and crown, extraction with or without subsequent replacement, or the option of no treatment. Based on the clinical assessment, the tooth has a favorable prognosis; thus, the indicated treatment option involves an endodontic treatment, succeeded by the placement of a post-core-crown restoration.

The endodontic treatment options and procedures were explained to the patient, including non-surgical root canal retreatment with either regenerative endodontic treatment (RET), conventional calcium hydroxide apexification, or one-step apexification. Following consultation with the prosthodontist, regenerative endodontic treatment was excluded due to the necessity of a post in the root canal space to support the ceramic crown; thus, one-step apexification was selected. Informed written consent was obtained from the patient to perform a one-step apexification procedure after engaging in a discussion regarding the treatment of the tooth. There was no ethical conflict.

### 2.1. First Treatment Visit

**Figure 2:**
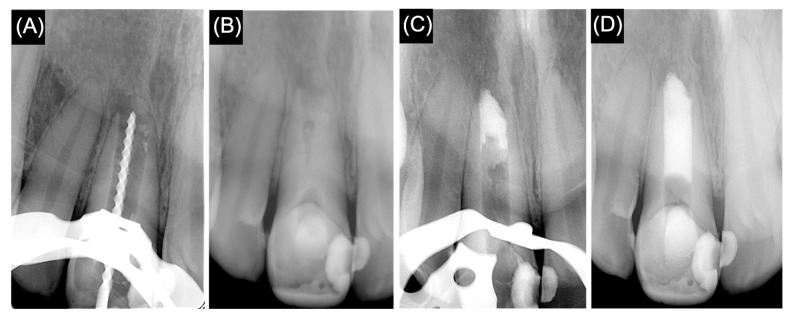
(**A**) Working length periapical radiograph. (**B**) Periapical radiograph after the placement of intracanal medicament. (**C**) Periapical radiograph showing the apical plug. (**D**) Final periapical radiograph.

The patient was anesthetized with 2% lidocaine with 1:100,000 epinephrine (Novocol Pharmaceutical, Cambridge, ON, Canada) using an infiltration technique. Tooth number 11 was isolated under a rubber dam, and the access cavity was re-opened. The gutta-percha was removed with H-files, and the working length was established using an electronic apex locater (Root ZX, J Morita MFQ Corp., Kyoto, Japan), measured at 0.5 mm short of the apex with a K-file #100 and confirmed by a radiograph (Figure 2A). The canal walls were not enlarged, and irrigation was conducted with 10 mL 1.5% sodium hypochlorite (NaOCl). A final flush with saline solution was performed, and the canal was dried with sterile paper points. Calcium hydroxide (UltraCal XS, Ultradent Products, Inc., South Jordan, UT, USA) medicament was placed in the root canal, then access cavity was sealed with temporary restorative material Cavit G (3M Deutschland GmbH, Seefeld, Germany) (Figure 2B), and the patient was given a second appointment 3 weeks later. All procedures were conducted under an operating microscope (ZEISS microscopy, Jena, Germany).

### 2.2. Second Treatment Visit

At the second visit, the patient was asymptomatic. Tooth number 11 was isolated using a rubber dam after the administration of local anesthetic, and access to the canal had been accomplished. The root canal was thoroughly irrigated with 10 mL of 1.5% NaOCl followed by a final rinse with 5 mL of saline solution, and then dried with sterile paper points. TotalFill^®^ BC RRM™ Putty (FKG Dentaire, Sàrl Le Crêt-du-Locle, Switzerland) was introduced into the canal and compacted apically using schilders pluggers (DENTSPLY Caulk, Milford, DE, USA). A periapical radiograph was exposed to confirm adequate placement of the apical plug (Figure 2C). The remaining part of the canal was backfilled with injectable thermoplasticized gutta-percha. Then, the access cavity was restored with a Ketac™ Molar-Aplicap glass ionomer (3M Deutschland GmbH, Seefeld, Germany) and light-cured composite Filtek™ Z350 XT (3M Deutschland GmbH, Seefeld, Germany). Subsequently, a final periapical radiograph was conducted (Figure 2D).

### 2.3. Follow-Up Visit

Clinical evaluation: The patient was recalled 6 months postoperatively, and after 2 years. The patient was asymptomatic during the follow up visits.

**Figure 3:**
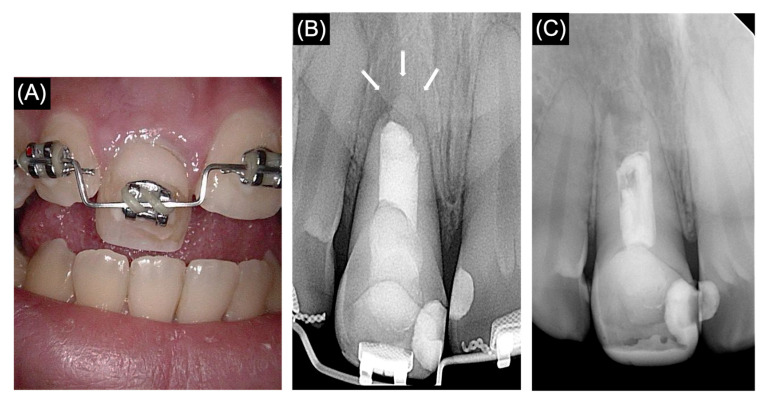
(**A**) Clinical photograph of tooth number 11 at the two-year follow-up visit. (**B**) Two-year follow-up periapical radiograph of tooth number 11. (**C**) Preoperative periapical radiograph of tooth number 11.

Radiographical evaluation: A two-year follow-up periapical radiograph showed the formation of a calcific barrier at the root apex with a normal periapical area in comparison to the preoperative periapical radiograph (Figure 3).

The objective assessment of the calcified bridge by radiographic imaging is as follows:

Calcified bridge dimension: the radiopaque band observed at the root apex demonstrates a sufficient thickness of approximately 2 mm in width and 3.5 mm in length, extending across the entire width of the canal to ensure an adequate apical closure.

Calcified bridge density: the radiopaque band exhibits uniformity, indicating consistent mineralization. Furthermore, the radiopacity is comparable to that of dentin or cementum and is clearly distinguishable from the surrounding radiolucent areas.

**Figure 4:**
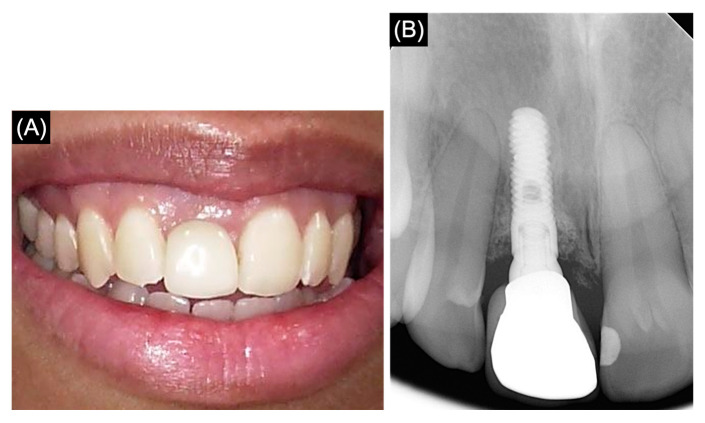
(**A**) Post-operative photograph of the restored single-tooth implant at site 11. (**B**) Periapical radiograph of the implant at site 11.

During the subsequent follow-up visits, I have been informed that the prosthodontic treatment plan has been revised, as the patient in this case preferred not to go for post-core crown restoration, and she preferred to place an implant for long-term survival. Consequently, the treatment option presented to the patient involved the continuation of endodontic therapy in conjunction with orthodontic extrusion to maintain the bone level before the implant placement. The orthodontic extrusion period lasted for 6 months, and the elastic was changed once a week. Subsequently, 3 months of stabilization were needed for the healing processes. Tooth #11 was replaced by a dental implant with a length of 10 mm and a width of 5 mm via a conventional protocol. The prosthetic part was made by using a PFM crown. A post-operative photograph and periapical radiograph of the restored single-tooth implant are shown in Figure 4.

### 2.4. Histologic Procedure

**Figure 5:**
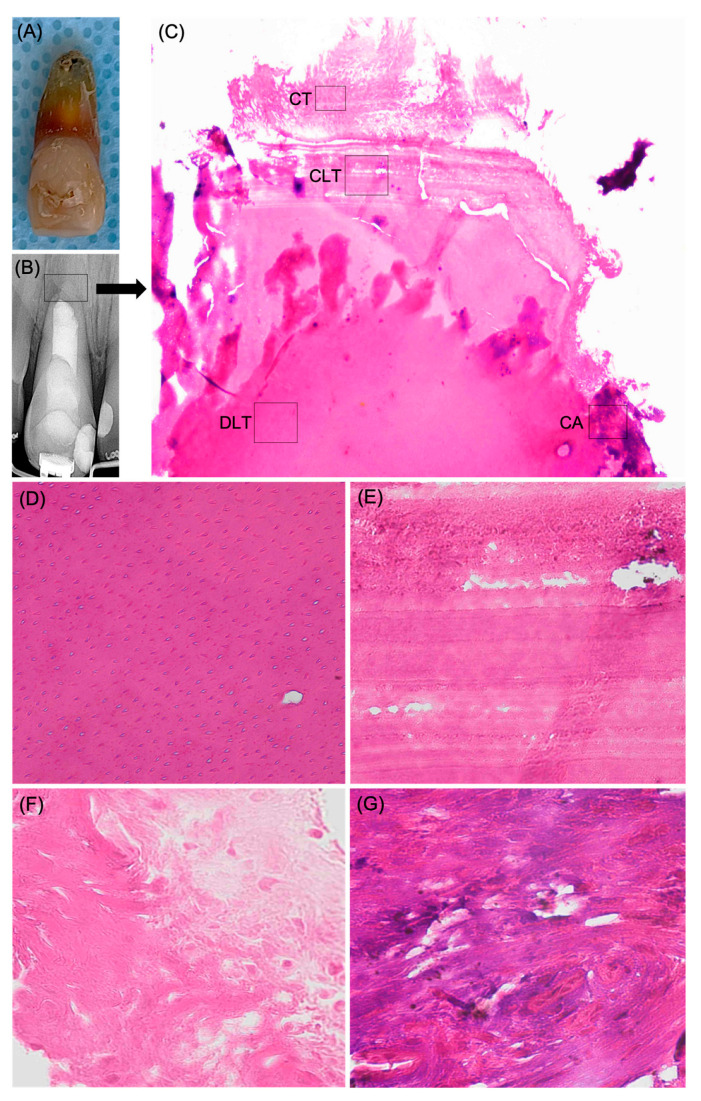
(**A**) A clinical photograph of the extracted tooth number 11. (**B**) Two-year follow-up periapical radiograph. (**C**) Hematoxylin-eosin staining image of the hard tissue formed at the root apex. Showing the dentin-like tissue (DLT) which is located adjacent to the apical plug filling material. Incremental layers of cementum-like tissue (CLT), which are most likely acellular cementum tissue, were formed adjacent to the dentin-like tissue. Connective tissue (CT) was observed next to the cementum-like tissue. Connective tissue with calcified areas (CA) were observed (original magnification ×4). (**D**) A higher-magnification view of the area indicated by the rectangle in image (**C**) of (DLT) dentin-like tissue, illustrating the dentinal tubule structures (original magnification ×40). (**E**) A higher-magnification view of the area indicated by the rectangle in image (**C**) of (CLT) cementum-like tissue, illustrating incremental deposition of acellular-like cementum tissue (original magnification ×40). (**F**) A higher-magnification view of the area indicated by the rectangle in image (**C**) of (CT) showing the connective tissue with distinct collagen fibers (original magnification ×40). (**G**) A higher-magnification view of the area indicated by the rectangle in image (**C**) of (CA), showing a connective tissue with areas of calcification (original magnification ×40).

Permission for histologic examination of the tooth was obtained from the patient. After extraction (Figure 5A), the tooth was immediately placed in a 10% neutral buffered formalin solution for fixation. After that, the tooth was decalcified in 7% formic acid until complete decalcification. Then the specimen was rinsed with running tap water for 2 hours, dehydrated with ascending concentrations of alcohol (70%, 90%, and 100%), and embedded in paraffin. After that, longitudinal serial sections were obtained with a microtome set at 4 µm thick in a buccolingual direction, and the specimens were stained with hematoxylin-eosin. Samples were observed under a light microscope to determine the histologic features.

### 2.5. Histologic Observation

The histologic findings showed the formation of mineralized tissue at the root apex (Figure 5C). The primary component of this recently developed apical barrier was a continuous layer of dentin-like tissue located adjacent to the apical plug, which was characterized by dentinal tubule structures (Figure 5D). Incremental layers of cementum-like tissue, which are most likely acellular cementum tissue, were formed adjacent to the dentin-like tissue (Figure 5E). Connective tissue with distinct collagen fibers was observed next to the cementum-like tissue (Figure 5F). Also, connective tissue with calcified areas were observed next to the dentin-like tissue (Figure 5G).

## 3. Discussion

This case report is presented in which mineralized apical tissue formation occurred in an endodontically failed maxillary central incisor with an open apex after the apexification procedure. The techniques used for managing the open apex in necrotic teeth, with or without apical periodontitis, went through many treatment phases, including conventional Ca(OH)_2_ apexification, artificial apical plug apexification, and regenerative endodontic treatment, each exhibiting various advantages as well as drawbacks. Conventional apexification using Ca(OH)_2_ has demonstrated reliable outcomes; however, several drawbacks have been noted, including the extended duration of treatment and the requirement for periodic replacement of the intracanal dressing, necessitating multiple visits and patient compliance. Additionally, there is an elevated risk of root fracture due to the prolonged presence of Ca(OH)_2_ within the root canal, as well as an increased likelihood of recontamination of the root canal system due to failures in the temporary seal [22,23]. To address these limitations, the artificial apical plug approach, referred to as one-step apexification, has been developed for managing such conditions. Nonetheless, this approach lacks the capacity to promote the thickening of canal walls and/or continued root growth [24,25].

The RET approach, unlike to the apexification procedure, promotes the growth of immature roots, involving root thickness and lengthening, apical closure, and potential regeneration of tooth vitality [26]. RET entails specific clinical considerations that must be adhered to in order to select the appropriate case. It is essential to consider patient and parental compliance, particularly given that the majority of cases involve young patients. Furthermore, it should be noted that the tooth does not necessitate the placement of a post or core within the pulp space, and the patient does not exhibit any allergies to the medications and antibiotics utilized in this procedure. While RET has indicated encouraging results, various limitations and adverse outcomes have been identified. This encompasses an extended treatment duration, numerous appointments for disinfection, variable histological results, possibility of crown discoloration, and the potential for treatment failure [27]. In this particular case, RET was excluded since the tooth was being designated for a post and core procedure. Consequently, one-step apexification has been selected as a treatment option.

The success of the apexification procedure depends on the deposition of the calcified barrier, which is controlled by the differentiation of the stem cells from the apical papilla (SCAP) that migrate from the healing periradicular tissues [28]. The molecular foundation of the apexification healing process involves various growth factors, cytokines, transcription factors, and bone morphogenetic proteins (BMPs) that facilitate the differentiation of SCAP into dentin-like, cementum-like, bone-like tissues, and/or organic matrix via specific signaling pathways [29]. The SCAP, derived from neural crest mesenchymal stem cells, are a distinct population with significant proliferative capacity, capable of self-renewal and exhibiting minimal immunogenicity [30]. Furthermore, the SCAP are capable of remaining viable in an infected immature permanent tooth with apical periodontitis, hence they are regarded as an essential biological source for the formation of the pulp-dentin complex and the continuing process of root development [31].

Prior histological studies indicated a variable response of apical tissue to the apexification procedure. An animal study conducted by Ham et al. demonstrated periapical healing and the formation of new calcified tissue, recognized as bone-like, cementum-like tissue, or osteodentin, at root apex of the infected, immature teeth [19]. An additional animal study by Palma et al. indicated that the developed apical barrier predominantly included cellular cementum encircled by periodontal ligament in most teeth treated with MTA apexification [21]. Yang et al. showed that the formed calcified barrier composed of immature hard tissue, connective tissue, and bone developed following calcium hydroxide apexification treatment of an immature human premolar tooth [20].

In this study, the histologic evaluation revealed the formation of an apical calcified barrier formed at the root apex, which was primarily composed of dentin-like tissue and cementum-like tissue. The dentin-like tissue located adjacent to the apical plug, distinguished by the presence of dentinal tubule structures. Subsequently, the incremental layers of cementum-like tissue were identified, possibly representing acellular cementum tissue. Furthermore, regions of connective tissue exhibiting distinct collagen fibers were noted, along with connective tissue containing calcified patches. We are unable to correlate our findings with the published data, which exhibit considerable variability in the type of newly formed tissue, likely attributable to differing study standards; some employed animal jaw models while others examined human teeth, alongside variations in treatment provided prior to histological assessment. Additionally, to the best of the author’s knowledge, this is the first histological study of an endodontically failed tooth that underwent successful apexification treatment.

The objective assessment of the calcified bridge enables clinicians to ascertain the effectiveness of the formed bridge in sealing the apex and supporting periapical healing. The specific characteristics of the calcified bridge, including size, dimension, and density, can be assessed using radiographic imaging techniques such as periapical radiography or cone beam computed tomography. The radiograph in this investigation indicated a radiopaque structure at the apex of the root canal, consistent with a mineralized barrier. The calcified bridge exhibits adequate dimensions, measuring approximately 2 mm in width and 3.5 mm in length. The density and radiographic characteristics indicate sufficient mineralization and closure of the apical foramen. These findings are consistent with previous studies reporting the formation of calcified barriers during apexification procedures [32,33,34,35].

Numerous biological factors that contribute to the failure of endodontic treatment have been identified. Nevertheless, the most prominent cause of failure is the persistence or regrowth of intraradicular infection [36]. The disinfection of root canal system in endodontically failed teeth is of great concern and may provide obstacles when managing an infected immature tooth with thin dentin walls when compared to their matured counterparts [37]. Evidence indicated that the use of Ca(OH)_2_ medicament in MTA apexification treatments considerably promoted periodontal tissue repair and regeneration. The majority of reported cases of apexification procedures, including the current report, were conducted through two clinical sessions, during which Ca(OH)_2_ was applied as an intracanal medicament [38].

The selection of material for the apical plug has a significant impact in the apexification outcomes. It must exhibit superior biocompatibility, facilitate stem cell migration and differentiation, possess antimicrobial properties, remain insoluble, be user-friendly, and not induce discoloration [4]. In addition to Ca(OH)_2_ and MTA materials, contemporary literature supports the use of calcium silicate bioceramic materials for apical barrier formation [39]. Interestingly, long-term prognostic studies demonstrated that apexification had high survival rates, irrespective of the type of bioactive material employed. High survival rates of Ca(OH)_2_ apexification have been reported to reach 86%, with an average follow-up duration of five years [40]. A recent long-term survival study of an immature traumatized incisors, indicated a median survival rate of 10 years for Ca(OH)_2_ apexification and 16 years with MTA apexification [41]. A retrospective study with an average follow-up duration of 3.3 years revealed that 86.3% of teeth treated with Biodentine^™^ as an apical plug exhibited complete healing or shown symptoms of healing [42].

A critical consideration in the treatment of teeth with wide-open apices is the avoidance of periapical extrusion of the apical plug filling material into the periradicular tissue. The excessive filling or extension of the apical filling material has been demonstrated in prior histological investigations to correlate with significant inflammatory cell infiltration and the lack of apical barrier tissue development [21,43]. This inflammatory process is thought to have impeded the repair of periodontal tissue, hence interfering with the formation of the hard tissue barrier. It has been recommended to employ a matrix at the periapex in wide-open apices to control the compaction of MTA material and prevent its extrusion. A variety of biocompatible materials have been documented in the literature for this purpose, including dentin chips, bovine bone xenografts, calcium phosphate, oxidized cellulose, and platelet-rich fibrin [44,45]. In the current study, we used a calcium silicate bioceramic material (TotalFill^®^ BC RRM™ Putty) as an apical plug, which is a pre-mixed condensable putty that allows for controlled administration without the necessity of an apical matrix.

An interdisciplinary approach, along with accurate diagnostics, is essential for achieving improved, conservative, and predictable outcomes in aesthetic areas. The endodontist performs a crucial role in advising patients regarding the decision-making process between tooth preservation and extraction. This encompasses a discussion of the advantages, risks, and long-term consequences related to each of the options [46]. In regard to the present case, endodontic therapy, succeeded by post-core-crown restoration, was identified as the preferred treatment modality. Nonetheless, in accordance with the patient’s preferences, the treatment plan was amended to accommodate extraction followed by implant replacement. Orthodontic extrusion is being implemented as a treatment modality that enhance both hard and soft tissue aspects prior to the implantation of dental implants [47]. The patient was satisfied with the color, morphology, and margins of the cemented restoration.

## 4. Conclusions

The present case demonstrates the clinical and radiographical success of an endodontically failed permanent incisor with an open apex after an apexification procedure. A two-year follow-up visit revealed the absence of signs and symptoms and hard tissue formation at the root apex. The histological evaluation of the newly formed mineralized tissue at the root apex revealed the formation of a continuous layer of dentin-like tissue with an identifiable dentinal tubule structure and the formation of an incremental layers of cementum-like tissue. In addition, connective tissue with distinct collagen fibers and connective tissue with calcified areas were noted.

## Data Availability

All data are contained within the article.
